# Sociocultural Reasons for Smokeless Tobacco Use Behavior

**DOI:** 10.5812/ijhrba.20002

**Published:** 2014-06-01

**Authors:** Leila Farhadmollashahi

**Affiliations:** 1 Department of Oral Medicine, Faculty of Dentistry, Zahedan University of Medical Sciences, Zahedan, IR Iran

**Keywords:** Culture, Social Environment, Tobacco Smokeless

Dear Editor

The use of smokeless tobacco, especially paan and gutka, is prevalent in Southeast Asian countries (1). No exact rate for the use of these products in Iran is available. According to the study by Honarmand et al. 11.4% of male students from universities of Zahedan City in Southeast of Iran were current users of smokeless tobacco (2). These products contain lime, areca-nut and tobacco (3). Evidence of the relationship between the use of the aforementioned ingredients and the following diseases has been reported: oral, throat and esophageal cancers, gum and tooth diseases, and dyspepsia (4), blood pressure, dyslipidemia, diabete, asthma, abortion and low birth weight (5).

Numerous social and cultural factors such as religious opinions, addiction, social popularity, and misassumptions about health benefits influence the use of smokeless tobacco (3). Some studies have reported the influence of religious activities on tobacco-induced behavior (6). The areca-nut content of tobacco has been introduced by some religions such as Hinduism as a divine fruit. They believe that this fruit is blessed by God and is distributed among his followers. Thus, they offer great deals of tobacco in ceremonies and weddings (3). On the other hand, the use of this substance is forbidden by many other religions (7). In some religions such as Islam, religious beliefs create a protective mechanism against stressful life events, which can accelerate the process of tobacco addiction (6, 8). In the study by Nakhaee et al. which aimed at investigating the relationship between religious activities and smoking in Iranian university students, a significant relationship was observed between the prevalence of smoking cigarettes and reading the Quran, praying, fasting and attending mosques (8).

Although Desalu introduces social acceptance as the main reason for the use of smokeless tobacco (4), conventions of Southeast Asian countries prohibit smoking for women and thus female smokers are known as “bad girls”. Therefore, women prefer to use smokeless tobacco. On the other hand, due to the prohibition of smoking in public places, consumers are encouraged to use smokeless tobacco (9).

Also, due to cultural, social and economic differences, ethnicity greatly influences the amount of consumption (10). Various studies have revealed that consumers of smokeless tobacco are not only unaware of the relationship between this product and oral cancer, but also believe that it has health benefits (3, 4). Therefore, some people use it as an astringent or means of oral cooling, cathartic, medicine for indigestion and for gynecological problems, intestinal parasitic infections, morning sickness, tooth decay, and bad breath. In addition, Friis et al. introduced other factors such as education, economic status, marital status, employment status and acculturation as factors influencing the prevalence of consumption of tobacco. Therefore, it seems that a plan for tobacco control should be focused on social and cultural factors of the consumer societies (9).
